# Combination HIV prevention during pregnancy and the post‐partum period in Malawi and Zambia: a mathematical modelling analysis

**DOI:** 10.1002/jia2.26128

**Published:** 2023-07-04

**Authors:** Kimberly A. Powers, Wilbroad Mutale, Nora E. Rosenberg, Lauren A. Graybill, Katie R. Mollan, Kellie Freeborn, Friday Saidi, Suzanne Maman, Priscilla L. Mulenga, Andreas Jahn, Rose K. Nyirenda, Jeffrey S. A. Stringer, Sten H. Vermund, Benjamin H. Chi

**Affiliations:** ^1^ Department of Epidemiology, Gillings School of Global Public Health The University of North Carolina at Chapel Hill Chapel Hill North Carolina USA; ^2^ School of Public Health University of Zambia Lusaka Zambia; ^3^ Department of Health Behavior, Gillings School of Global Public Health The University of North Carolina at Chapel Hill Chapel Hill North Carolina USA; ^4^ Department of Obstetrics and Gynecology, School of Medicine The University of North Carolina at Chapel Hill Chapel Hill North Carolina USA; ^5^ UNC Project Malawi Lilongwe Malawi; ^6^ Directorate of Public Health Zambia Ministry of Health Lusaka Zambia; ^7^ Department of HIV and AIDS Malawi Ministry of Health Lilongwe Malawi; ^8^ International Training and Education Center for Health (I‐TECH), Department of Global Health University of Washington Seattle Washington USA; ^9^ Department of Epidemiology of Microbial Diseases Yale School of Public Health New Haven Connecticut USA

**Keywords:** Africa, infectious disease transmission, perinatal care, prenatal care, sexual behaviour, sexual partners

## Abstract

**Introduction:**

Despite widespread success in reducing vertical HIV transmission, most antenatal care (ANC) programmes in eastern and southern Africa have not emphasized primary prevention of maternal HIV acquisition during pregnancy and lactation/breastfeeding. We hypothesized that combination HIV prevention interventions initiated alongside ANC could substantially reduce maternal HIV incidence.

**Methods:**

We constructed a multi‐state model describing male‐to‐female HIV transmission in steady heterosexual partnerships during pregnancy and lactation/breastfeeding, with initial conditions based on population distribution estimates for Malawi and Zambia in 2020. We modelled individual and joint increases in three HIV prevention strategies at or soon after ANC initiation: (1) HIV testing of male partners, resulting in HIV diagnosis and less condomless sex among those with previously undiagnosed HIV; (2) initiation (or re‐initiation) of suppressive antiretroviral therapy (ART) for male partners with diagnosed but unsuppressed HIV; and (3) adherent pre‐exposure prophylaxis (PrEP) for HIV‐negative female ANC patients with HIV‐diagnosed or unknown‐status male partners. We estimated the percentage of within‐couple, male‐to‐female HIV transmissions that could be averted during pregnancy and lactation/breastfeeding with these strategies, relative to base‐case conditions in which 45% of undiagnosed male partners become newly HIV diagnosed via testing, 75% of male partners with diagnosed but unsuppressed HIV initiate/re‐initiate ART and 0% of female ANC patients start PrEP.

**Results:**

Increasing uptake of any single strategy by 20 percentage points above base‐case levels averted 10%−11% of maternal HIV acquisitions during pregnancy and lactation/breastfeeding in the model. Joint uptake increases of 20 percentage points in two interventions averted an estimated 19%−23% of transmissions, and with a 20‐percentage‐point increase in uptake of all three interventions, 29% were averted. Strategies achieving 95% male testing, 90% male ART initiation/re‐initiation and 40% female PrEP use reduced incident infections by 45%.

**Conclusions:**

Combination HIV prevention strategies provided alongside ANC and sustained through the post‐partum period could substantially reduce maternal HIV incidence during pregnancy and lactation/breastfeeding in eastern and southern Africa.

## 1 INTRODUCTION

The rising uptake of lifelong antiretroviral therapy (ART) among pregnant people living with HIV and attending antenatal care (ANC) resulted in a 38% reduction in vertical HIV transmission between 2010 and 2019 [1]. Far less attention has been directed towards structured, ANC‐based interventions to prevent maternal HIV acquisition during pregnancy and lactation/breastfeeding. In hyper‐endemic sub‐Saharan African settings, HIV incidence during pregnancy and lactation/breastfeeding is as high as 2.1/100 person‐years [2], with as many as 120,000 pregnant or lactating/breastfeeding people acquiring HIV across 21 UNAIDS focus countries in 2020 [3]. HIV infections acquired during these periods affect maternal health [4, 5] and have contributed to a growing proportion of infant HIV infections in the Option B+ era [3], hindering efforts to eliminate vertical HIV transmission.

ANC settings provide unique opportunities for the prevention of heterosexual HIV acquisition, given increased healthcare engagement [6], greater partner involvement [7] and motivation to protect the unborn child during this period [8]. We previously introduced a conceptual framework for partner‐based, combination HIV prevention strategies extending from antenatal services [9]. With this framework, we argued that health system knowledge of a pregnant patient's HIV status—alongside that of her partner—can guide primary HIV prevention for the expectant parents. For reductions in male‐to‐female HIV transmission, our framework focused on three prevention approaches during pregnancy and lactation/breastfeeding: (1) increased male partner HIV testing to improve HIV status awareness and reduce transmission risk behaviours; (2) increased support for ART initiation and adherence among male partners living with HIV; and (3) introduction of pre‐exposure prophylaxis (PrEP) for HIV‐negative ANC patients with partners known to be living with HIV or with unknown HIV status. This framework has guided our ongoing work in Malawi and Zambia, including formative research [2, 10, 11, 12] and pilot trials [13, 14]. In this study, we use mathematical modelling to estimate the potential impact of these HIV prevention approaches on within‐couple, male‐to‐female HIV transmission among pregnant and lactating/breastfeeding people attending ANC in these countries.

## 2 METHODS

### 2.1 Model structure and initial conditions

**Figure 1:**
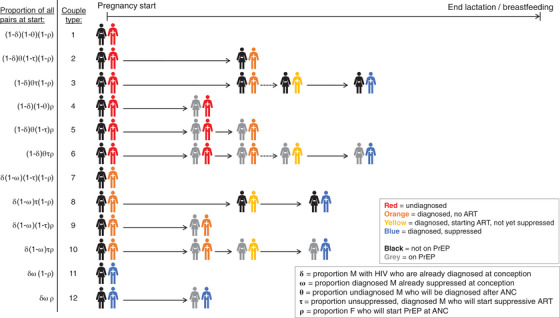
Model diagram: male‐to‐female HIV transmission within steady partnerships in which the pregnant female attends ANC services. The model includes 12 couple types (numbered 1–12) defined by: (a) the male partner's initial and subsequent HIV diagnosis and viral suppression status, and (b) the female partner's eventual PrEP status. The equations on the far left specify the proportion of couples initiated as each couple type, and the timeline from left to right (not drawn to scale) delineates changes to PrEP, diagnosis and viral suppression status over the course of pregnancy and lactation/breastfeeding. Not pictured is male‐to‐female transmission within couples, which is modelled as a function of: (a) condomless sexual contact rates, which are assumed to decrease upon diagnosis; and (b) per‐act HIV transmission risk, which is assumed to decrease in the presence of PrEP and/or suppressive ART. Transmission brings couples to an HIV‐concordant‐positive absorbing state in the model; all such events are tracked to allow calculation of HIV incidence during pregnancy and lactation/breastfeeding. Dashed lines indicate transitions occurring in the model on the same day as the immediately prior transition (i.e. same‐day ART initiation upon HIV diagnosis).

As detailed in the Supplementary Material, we constructed a multi‐state model describing HIV transmission during pregnancy and lactation/breastfeeding within steady, heterosexual pairs comprising a male with HIV and a female without HIV at the start of pregnancy (Figure 1). We initiated the model at zero gestational days, and we tracked couples over time in terms of the female partner's PrEP and HIV infection status and the male partner's HIV diagnosis and viral suppression status. We considered a hypothetical population of 12 million such couples, and we based the initial distribution of couples across HIV status/diagnosis/suppression categories on national, population‐based estimates from Malawi and Zambia [15, 16, 17] (Table 1). To analyse transmission occurring in different time intervals, we ran the model through four different endpoints for all couples: (1) 91 days (through early pregnancy [18]); (2) 280 days (through full‐term pregnancy); (3) 449 days (through an early post‐partum period of 24 weeks [18]); and (4) 916 days (through a lactation/breastfeeding period of 636 days, the midpoint of median estimates for Malawi and Zambia [15, 16]).

**Table 1:** Initial conditions and base‐case input parameter values

Description	Malawi	Zambia	Composite^a^	Source(s)
*Initial conditions (at pregnancy start)*:				
Proportion male partners with HIV who are aware of HIV status (δ)	0.86	0.87	0.87	[17]
Proportion HIV‐diagnosed male partners with viral suppression (ω)	0.73	0.81	0.77	[17]
Proportion HIV‐negative females on PrEP	0	0	0	[17]
*Intervention‐related input parameters*:				
Time of first ANC visit (weeks of gestation)	21	19	20	[15, 16]
Proportion HIV‐negative females initiating PrEP at first ANC visit (ρ)			0^b^	[27]
Proportion undiagnosed males with HIV becoming diagnosed via testing after first ANC visit (θ)			0.45^b^	[13]
Proportion newly or previously HIV‐diagnosed males starting suppressive ART after first ANC visit (τ)			0.75^b^	[17]^c^
*Transmission parameters*:				
Monthly number of condomless sexual acts when partner with HIV is unaware of HIV status:				
Early pregnancy (Days 0–91)			4.71	[18, 22]
Late pregnancy (Days 92–280)			3.24	[18, 22]
Early post‐partum (First 24 weeks after end of full‐term pregnancy = Days 281–449)			2.32	[18, 22]
Late post‐partum (Days 450–916)			4.62	[18, 22]
Per‐act heterosexual HIV transmission risk in the absence of condoms, PrEP and suppressive ART:				
Early pregnancy (Days 0–91)			0.0022	[18]
Late pregnancy (Days 92–280)			0.0030	[18]
Early post‐partum (First 24 weeks after end of full‐term pregnancy = Days 281–449)			0.0042	[18]
Late post‐partum (Days 450–916)			0.0011	[18, 23]
Relative frequency of condomless sexual acts when partner with HIV is status‐aware (vs. unaware)			0.47	[24]
Relative per‐act risk of heterosexual HIV transmission in the presence (vs. absence) of suppressive ART			0.04	[26]
Relative per‐act risk of heterosexual HIV transmission in the presence (vs. absence) of adherent PrEP			0.25	[25]

^a^ Used in model.

^b^ Key intervention uptake parameter varied in modelling scenarios. Base‐case values shown here.

^c^ Assumed value of 0.75 was chosen to approximate cross‐sectional, population‐based estimates of 0.73−0.81 for the proportion of all diagnosed males with viral suppression in Malawi and Zambia in [17].

To reflect the near‐universality of ANC [15, 16] and ANC‐based HIV testing [19] in both countries, we assumed that the female member of each couple accessed ANC at least once during pregnancy and received HIV testing at that visit. Based on estimates of ANC initiation timing from Malawi and Zambia [15, 16], this visit was assumed to occur at 140 days (20 weeks) of gestation. Patients could also initiate oral PrEP as once‐daily tenofovir‐emtricitabine at this visit, with uptake varying across model scenarios. In the main analysis, we assumed that once initiated, adherent PrEP use was maintained throughout pregnancy and lactation/breastfeeding, an assumption we probed in sensitivity analyses (see below).

Our model described HIV testing and treatment among male partners via two discrete parameters. The first parameter represented male HIV testing, which we defined as the proportion of previously undiagnosed male partners with HIV who newly accessed HIV testing after the female partner attended ANC. The second parameter reflected the proportion of virologically unsuppressed male partners (both newly and previously diagnosed) who initiated (or re‐initiated) suppressive ART after female ANC presentation. HIV testing (for previously undiagnosed partners) and suppressive ART initiation (for both newly and previously diagnosed partners) were assumed to occur 15 days after the female ANC visit [13], and viral suppression was assumed to begin 30 days after ART initiation to approximate the rapid viral load reductions observed upon ART initiation in randomized trials [20, 21]. Male suppressive ART use was assumed in the main analysis to continue through pregnancy and lactation/breastfeeding, another assumption we probed in sensitivity analyses (see below).

We modelled male‐to‐female HIV transmission within partnerships as a function of condomless sexual contact rates and per‐contact transmission probabilities [18, 22, 23], both of which varied over the course of pregnancy and the post‐partum period [18] (Table 1). HIV diagnosis among male partners reduced condomless sexual contact rates [24] in the model, and both viral suppression (in males) and PrEP use (by females) reduced per‐contact transmission probabilities [25, 26]. Relative reductions in transmission probabilities were assumed to be multiplicative when two or more prevention strategies (e.g. PrEP and suppressive ART) were acting.

### 2.2 Base‐case and intervention scenarios

We first simulated a “base‐case” scenario designed to reflect the current uptake of the three prevention strategies of interest: (1) HIV testing/diagnosis among undiagnosed male partners after female ANC presentation; (2) incident suppressive ART uptake among HIV‐diagnosed, virologically unsuppressed male partners after female ANC presentation; and (3) PrEP uptake at first ANC among HIV‐negative pregnant patients whose male partners were living with diagnosed HIV or had unknown HIV status. In this base‐case scenario, we assumed that 45% of previously undiagnosed male partners became newly aware of their HIV status via testing, 75% of unsuppressed male partners with previously or newly diagnosed HIV initiated (or reinitiated) ART and 0% of females initiated PrEP. The base‐case value for male testing was based on formative work we conducted among HIV‐negative patients presenting to ANC in Zambia before we launched a pilot randomized trial [13] of partner HIV testing strategies in this setting. The base‐case value for PrEP uptake among HIV‐negative patients was based on recent service delivery estimates in Malawi and Zambia [27]. In the absence of reliable empirical estimates for male partner ART uptake after female ANC presentation, we selected the base‐case value of 75% for incident viral suppression among male partners to approximate cross‐sectional, population‐based estimates of viral suppression among all diagnosed males in Malawi and Zambia [17]. Given the uncertainty around this value, we conducted sensitivity analyses (see below) with lower and higher base‐case values.

We then simulated a range of intervention scenarios in which we singly and jointly increased uptake of the three HIV prevention strategies, with male HIV testing and ART uptake ranging up to 100% and female PrEP uptake to 40%. We assessed the HIV prevention impact of these three intervention strategies, both alone and in combination, by estimating the percentage of within‐couple, male‐to‐female HIV transmissions averted in each scenario, relative to the base case.

In addition to examining this full range of uptake combinations, we defined three sets of discrete scenario types: one in which uptake of one, two or all three strategies was increased by 10 percentage points; one in which uptake was increased by 20 percentage points; and one in which uptake was increased to strategy‐specific targets. In the last (“target”) scenario, we assumed that male testing was increased to 95%, suppressive ART uptake among diagnosed male partners was increased to 90% and PrEP uptake among HIV‐negative ANC patients with diagnosed or undiagnosed partners with HIV (i.e. all ANC patients in the model) was increased to 40%. The targets for male testing and suppressive ART were chosen to align with UNAIDS 95‐95‐95 goals [27], whereby 95% of people with HIV should be diagnosed, 95% of persons with diagnosed HIV should initiate ART and 95% of those on ART should be virally suppressed (i.e. 95% × 95% = 90% of all HIV‐diagnosed persons should be suppressed on ART). The female PrEP target was based on conditions observed in a public health setting in Kenya [28].

### 2.3 Sensitivity analyses

To assess the sensitivity of results to key model assumptions, we conducted five sets of sensitivity analyses. In the first set, we repeated our analyses with assumed base‐case values of 60% and 80% (rather than 75%) for ART initiation/re‐initiation among diagnosed male partners. In the second, we assumed a constant per‐act HIV transmission probability in the absence of condoms, ART, and PrEP throughout pregnancy and lactation/breastfeeding. We fixed this transmission probability at 0.002, representing the weighted average (by time spent in each sub‐interval) of the time–varying values assumed in the main analysis. In the third set of sensitivity analyses, we varied the assumed effectiveness of each HIV prevention strategy by increasing or decreasing the risk ratio corresponding to its impact on the per‐act transmission risk (for female PrEP and male ART) or the frequency of condomless coital acts (for HIV testing/diagnosis) to reflect potential effectiveness differences in our population versus those in which the main analysis values of these input parameters were derived. In this third set, we modelled eight scenarios: one in which all intervention‐associated transmission risk ratios were multiplied by 1.2 (reflecting a 20% relative decrease in effectiveness vs. main analysis assumptions), one in which all three risk ratios were multiplied by 0.8 (reflecting a 20% relative effectiveness increase), three in which one risk ratio was multiplied by 0.8 and the other two by 1.2, and three in which one risk ratio was multiplied by 1.2 and the other two by 0.8.

In the fourth set of sensitivity analyses, we allowed for lower PrEP adherence (and thus effectiveness) among ANC patients whose male partners were on suppressive ART (vs. those whose partners were not on ART) by multiplying the transmission risk ratio associated with PrEP use by a factor of 1.5 (reflecting a 50% effectiveness decrease) in these couples. Finally, in the fifth set of sensitivity analyses, we allowed for lower intervention adherence/effectiveness in the post‐partum period by multiplying transmission risk ratios during this period by a factor of 1.5 in all couples with incident male diagnosis, male viral suppression and/or female PrEP use during pregnancy.

All modelling analyses were conducted with the gems package [29] using R Version 3.6.3 [R Core Team, 2020].

## 3 RESULTS

In the base‐case scenario, within‐couple male‐to‐female cumulative HIV incidence was 0.7% in early pregnancy, 1.7% at the end of full‐term pregnancy, 2.3% after 6 post‐partum months, and 3.1% after the full period of pregnancy and lactation/breastfeeding (Table 2). These cumulative incidence values corresponded to an overall HIV incidence rate of 1.3/100 person‐years during pregnancy and lactation/breastfeeding in this model population of steady mixed‐HIV‐status partnerships. Interval‐specific incidence rates in the full model population ranged from 3.0/100 person‐years in early pregnancy to 0.6/100 person‐years in the late post‐partum period. Within sub‐cohorts in which the female did not start PrEP, overall HIV incidence rates were 9.0/100 person‐years in couples where the male partner remained undiagnosed (i.e. “type 1” couples in Figure 1), 4.2/100 person‐years in couples where the male partner had been diagnosed before conception but did not initiate/re‐initiate ART (i.e. “type 7” couples) and 0.2/100 person‐years in couples where the male partner was on ART from the start (i.e. “type 11” couples).

**Table 2:** Model‐estimated maternal HIV incidence by sub‐interval of pregnancy or lactation/breastfeeding

		Cumulative HIV incidence at the end of period^a^, ^b^ Point estimate (95% CI)				HIV incidence rate during period^b^, ^c^ Point estimate				
Couple type^d^	Base‐case % of couples	Early pregnancy	Full‐term pregnancy	Early post‐partum	Lactation/breastfeeding	Early pregnancy	Late pregnancy	Early post‐partum	Late post‐partum	Full 916‐day period
1	7.1	3.1 (2.7,3.4)	8.8 (8.3,9.4)	13.7 (13.1,14.3)	20.3 (19.4,21.0)	12.6	11.4	10.8	5.3	9.0
2	1.5	3.1 (2.7,3.4)	6.9 (6.4,7.3)	9.2 (8.7,9.8)	12.5 (11.9,13.2)	12.6	7.4	5.2	2.6	5.3
3	4.4	3.1 (2.7,3.4)	5.5 (5.0,6.0)	5.6 (5.1,6.1)	5.8 (5.3,6.2)	12.6	4.8	0.2	0.1	2.4
4	0.0	3.1 (2.7,3.4)	5.7 (5.2,6.1)	7.0 (6.5,7.5)	8.8 (8.3,9.3)	12.6	5.1	2.8	1.4	3.7
5	0.0	3.1 (2.7,3.4)	5.2 (4.7,5.7)	5.8 (5.4,6.3)	6.7 (6.2,7.2)	12.6	4.1	1.3	0.7	2.8
6	0.0	3.1 (2.7,3.4)	4.9 (4.4,5.3)	4.9 (4.4,5.3)	4.9 (4.4,5.4)	12.6	3.5	0.1	0.03	2.0
7	5.0	1.5 (1.3,1.7)	4.3 (3.9,4.6)	6.7 (6.2,7.1)	10.1 (9.6,10.7)	5.9	5.5	5.3	2.7	4.2
8	15.0	1.5 (1.3,1.7)	2.9 (2.6,3.2)	3.0 (2.7,3.3)	3.1 (2.8,3.5)	5.9	2.8	0.2	0.1	1.3
9	0.0	1.5 (1.3,1.7)	2.7 (2.4,3.0)	3.3 (3.0,3.7)	4.2 (3.9,4.7)	5.9	2.5	1.3	0.7	1.7
10	0.0	1.5 (1.3,1.7)	2.4 (2.1,2.6)	2.4 (2.1,2.7)	2.4 (2.2,2.7)	5.9	1.8	0.1	0.03	1.0
11	67.0	0.06 (0.01,0.1)	0.2 (0.1,0.3)	0.3 (0.2,0.4)	0.4 (0.3,0.5)	0.2	0.2	0.2	0.1	0.2
12	0.0	0.06 (0.01,0.1)	0.1 (0.06,0.2)	0.1 (0.07,0.2)	0.2 (0.1,0.3)	0.2	0.1	0.1	0.03	0.07
Overall	100.0	0.73 (0.68,0.78)	1.74 (1.65,1.83)	2.33 (2.22,2.44)	3.14 (3.02,3.26)	3.0	2.0	1.3	0.6	1.3

^a^ Percentage of ANC patients without HIV at pregnancy start who acquire HIV in the model by end of specified period.

^b^ Early pregnancy = first 91 days after start of pregnancy; late pregnancy = days 92–280; early post‐partum = days 281–449; late post‐partum = days 450–916 [18]. Full‐term pregnancy = early + late pregnancy. Lactation/breastfeeding = early + late post‐partum.

^c^ Rate of incident maternal HIV infections per 100 person‐years at risk during a given period, calculated from model‐estimated cumulative incidence and the exponential formula (cumulative incidence proportion at end of interval of duration *t* years = 1 – e^−r*^ *^t^*, where *r* = rate per person‐year).

^d^ See Figure 1.

**Figure 2:**
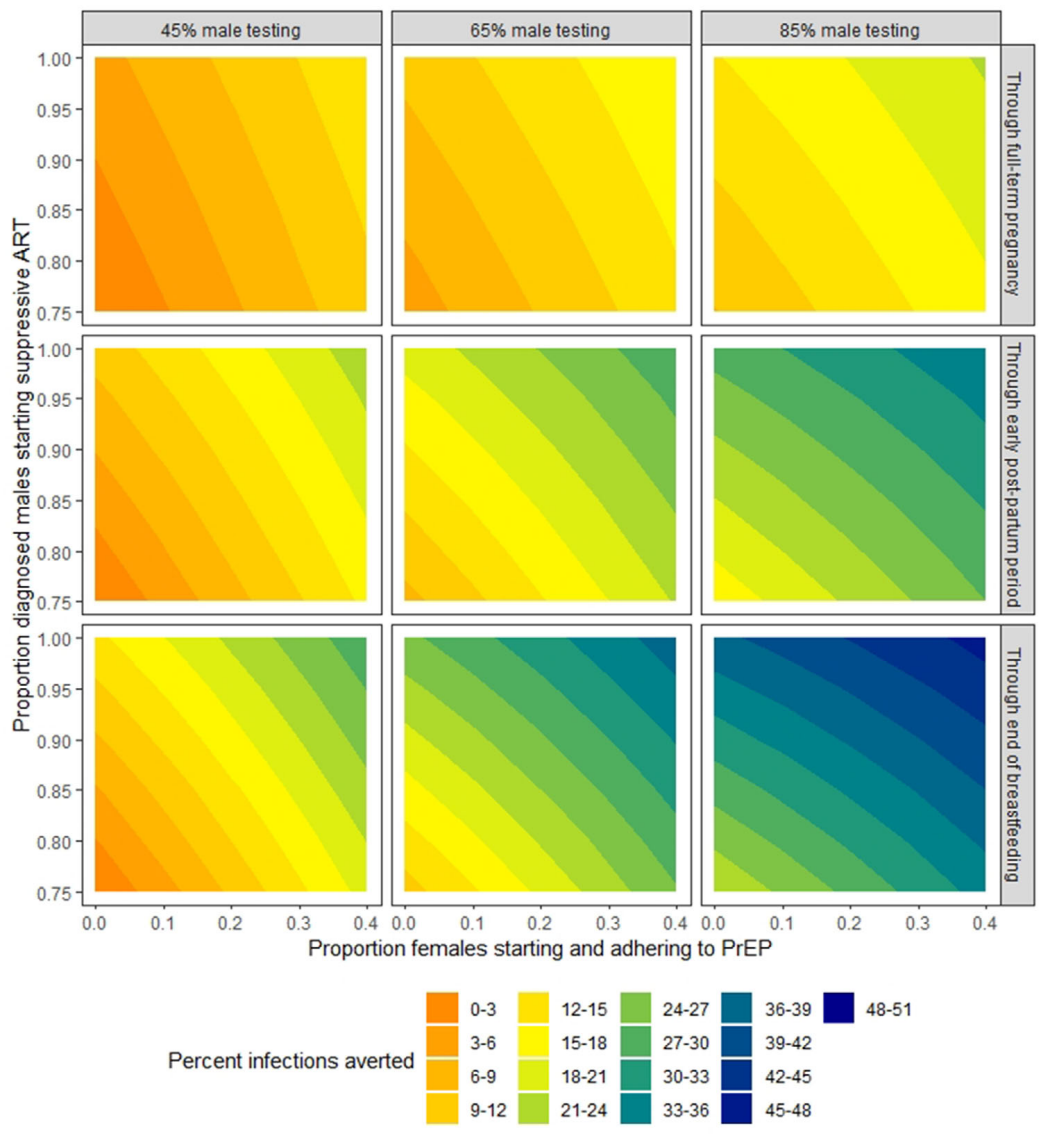
Estimated intervention impact (percent of infections averted) across range of scenarios. Shaded region represents the percentage of within‐couple, male‐to‐female HIV infections averted during the specified intervals of pregnancy and lactation/breastfeeding (compared to base‐case conditions) according to the proportion of HIV‐negative female patients starting PrEP at the first ANC visit (horizontal axis) and the proportion of virologically unsuppressed, HIV‐diagnosed male partners initiating/re‐initiating suppressive ART after the first ANC visit (vertical axis) in scenarios where (left to right): 45% of partners become newly diagnosed through testing (base case), 65% of partners become newly diagnosed through testing and 85% of partners become newly diagnosed through testing. Because all interventions begin at or after ANC initiation, which occurs after the end of early (first‐trimester) pregnancy, the impact of all interventions in the first 91 days is zero (and thus not shown).

Increasing uptake of any of the three prevention strategies resulted in greater numbers of heterosexual transmissions averted in the model, with transmissions averted growing over time (Figure 2). For example, increasing female PrEP uptake from 0% to 20% resulted in a 5.5% (95% confidence interval [CI]: 0.0%–11.8%) relative reduction of within‐couple incident HIV infections over 280 days of gestation, and a 9.6% (95% CI: 5.1%–14.1%) reduction over the full 916‐day simulation. When PrEP uptake was increased from 0% to 40%, an estimated 11.0% (95% CI: 5.4%–16.6%) of infections were averted through 280 days, and 19.2% (95% CI: 15.3%–23.0%) through the end of lactation/breastfeeding. These impacts increased with concomitant increases of incident viral suppression among diagnosed male partners. For example, with an increase of 20 percentage points in male viral suppression added to the same increase in female PrEP, infections averted increased to 19.3% (95% CI: 15.0%–23.6%) after 916 days, compared to the 9.6% predicted with a 20‐percentage‐point increase in PrEP alone. As male partner testing increased (moving left to right across Figure 2 panels), the impact of both female PrEP and male ART uptake also increased, particularly when both strategies were scaled up in combination. For instance, when male partner testing was 65% in the model, an estimated 18.6% (95% CI: 14.5%–22.8%) of HIV infections were averted (vs. the base case) through 916 days when female PrEP uptake was 20% and suppressive ART uptake among diagnosed males was 75%. This estimate increased to 29.3% (95% CI: 25.3%–33.2%) when suppressive ART uptake among diagnosed male partners was increased to 95%. We observed similar trends in sensitivity analyses where base‐case suppressive ART uptake was 60% (Figure S1) or 80% (Figure S2), although the effects of increasing ART uptake among diagnosed male partners were more pronounced at lower base‐case values. Trends were again similar when we assumed a fixed per‐act transmission probability throughout pregnancy and lactation/breastfeeding (Figure S3), although estimated intervention impacts were slightly lower during pregnancy and the early post‐partum period, and slightly higher over the full simulation period (i.e. through lactation/breastfeeding), than in the main analysis.

**Figure 3:**
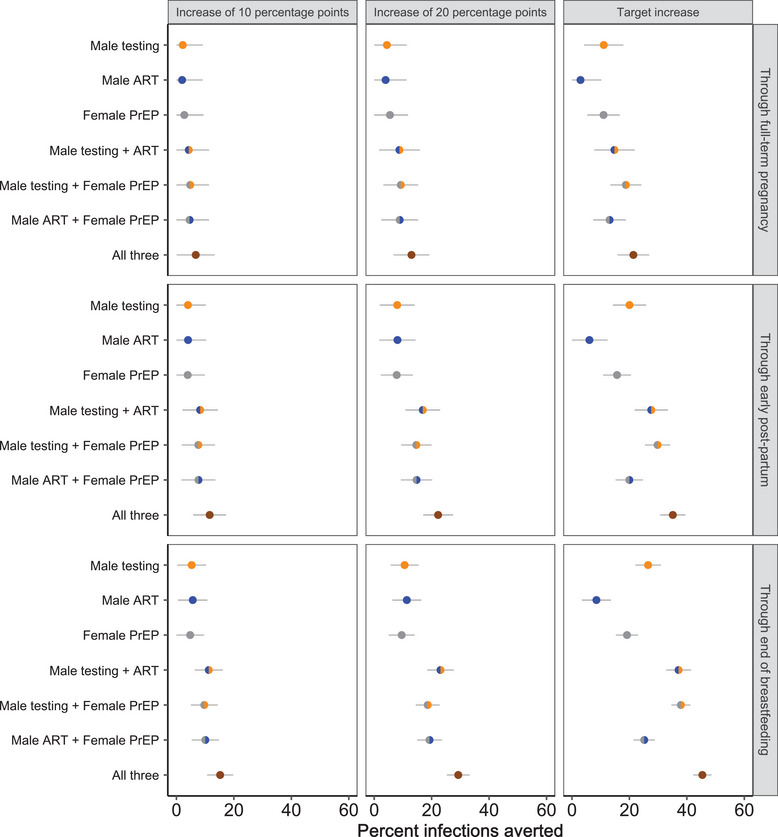
Estimated intervention impact (percent infections averted) in discrete intervention scenarios. Circles represent the percentage of intra‐couple, male‐to‐female HIV infections averted during the specified intervals of pregnancy and lactation/breastfeeding (compared to base‐case conditions) according to discrete intervention scenarios comprising one, two and all three interventions of interest. Gold represents male HIV testing, blue represents male ART initiation/re‐initiation, grey represents female PrEP and brown represents a combination of all three interventions. Horizontal lines represent 95% confidence intervals. In the “target increase” scenarios, male HIV testing uptake is increased to 95% (from a base‐case value of 45%), male ART initiation/re‐initiation is increased to 90% (from a base‐case value of 75%) and female PrEP uptake is increased to 40% (from a base‐case value of 0%).

When uptake of each intervention was separately increased by 10 percentage points, an estimated 2.0%−2.7% of infections were averted (relative to the base case) through 280 days and 4.8%−5.7% through 916 days (single‐coloured circles, left column of panels in Figure 3). Joint increases of 10 percentage points in each combination of two interventions resulted in 4.3%−4.8% of transmissions averted through 280 days and 9.7%−11.3% through 916 days (dual‐coloured circles, Figure 3). An increase of 10 percentage points in all three interventions resulted in an estimated 6.7% (95% CI: 0.1%–13.4%) of infections averted through 280 days and 15.2% (95% CI: 10.7%–19.8%) through 916 days (brown circles, Figure 3). Increases of 20 percentage points resulted in greater estimated transmission impacts than those attained with increases of 10 percentage points (cf. middle and left column of panels in Figure 3), with single interventions resulting in transmission reductions of 9.6%−11.4%, two‐intervention combinations resulting in transmission reductions of 18.6%−23.1% and three‐intervention combinations resulting in transmission reductions of 29.3% (95% CI: 25.3%–33.2%) through 916 days. Increasing uptake of single interventions to “target” levels resulted in estimated transmission impacts of 8.5%−26.5% at 916 days, and joint increases to target levels of two interventions resulted in reductions of 25.2%−37.9% (right column of panels in Figure 3). Reaching target uptake for all three interventions resulted in estimated transmission reductions of 45.4% (95% CI: 42.2%–48.6%) through 916 days.

**Figure 4:**
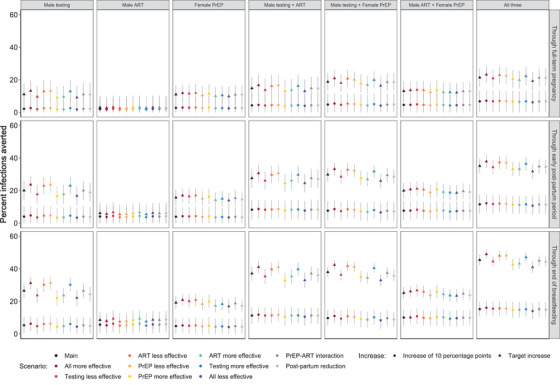
Comparison of main and sensitivity analyses. Model‐predicted percentages of incident HIV infections averted by single‐, double‐ and triple‐intervention combinations in the main analysis (black symbols) and sensitivity analyses (coloured symbols for assumptions of +/− 20% in intervention effectiveness; dark grey symbols for reduced PrEP adherence/effectiveness in female ANC patients with male partners on ART [“PrEP‐ART interaction”]; light grey symbols for reduced PrEP/ART adherence in the post‐partum period among incident PrEP/ART users [“Post‐partum reduction”]). For scenarios labelled as having one intervention that was more effective than in the main analysis, the other two interventions were less effective than in the main analysis. For scenarios labelled as having one intervention that was less effective than in the main analysis, the other two interventions were more effective than in the main analysis. Circles represent scenarios where intervention coverage was increased 10 percentage points; triangles represent increases to “target” levels.

Overall findings with respect to the relative impacts of different intervention combinations were similar in sensitivity analyses where we varied assumptions about intervention effectiveness (Figure 4). As in the main analysis, for any given sensitivity scenario, two‐intervention combinations were predicted to be more effective than single interventions, and a three‐way combination of male testing, male viral suppression and female PrEP was predicted to have the greatest impact. Across all sensitivity analysis scenarios, the percentage of infections averted by a given intervention (or a combination thereof) was within 5 percentage points of the corresponding main analysis estimate, with the greatest differences relative to the main results occurring in scenarios where target uptake levels were assumed.

## 4 DISCUSSION

More than a decade after Option B+ was introduced in Malawi and then scaled across the African continent, maternal HIV incidence remains high and elimination of vertical HIV transmission has not been achieved. There is thus a pressing need to identify effective approaches for preventing maternal HIV acquisition during pregnancy and lactation/breastfeeding. Our modelling indicates that with sufficient uptake and sustained adherence, combination HIV prevention strategies focused on male partner HIV testing, male partner ART, and/or female PrEP during pregnancy and lactation/breastfeeding could substantially reduce HIV acquisition among patients receiving ANC in eastern and southern Africa.

Estimated incidence reductions arising solely from increased male HIV testing varied across scenarios, with the greatest reduction predicted in circumstances where the target level of 95% uptake was achieved. When increases in male HIV testing were coupled with increases in male ART uptake, synergies between the two interventions produced appreciable transmission impacts, as testing/diagnosis not only reduced condomless sex in the model, but also enabled ART initiation. The synergistic effects of joint intervention increases were particularly apparent when target levels of both interventions were reached and the full period of pregnancy and lactation/breastfeeding had concluded, underscoring the importance of both high initial uptake and long‐term adherence.

As with male partner HIV testing, increasing adherent PrEP uptake to a target of 40% among ANC patients whose partners were living with diagnosed or undiagnosed HIV had a substantial predicted impact. This potential for effective maternal PrEP to substantially reduce HIV incidence echoes the findings of a modelling study of PrEP among pregnant and lactating/breastfeeding people in South Africa [30]; however, the prior study reported overall HIV incidence reductions, rather than male‐to‐female incidence alone, hindering direct comparison.

Our base‐case estimate of maternal HIV incidence in mixed‐HIV‐status couples during pregnancy and lactation/breastfeeding (1.3/100 person‐years) is similar to the summary estimate of our recent meta‐analysis [2], in which estimated HIV incidence during pregnancy and lactation/breastfeeding in sub‐Saharan Africa (without specification of partner infection, diagnosis or treatment status) was 2.1 (95% CI: 0.7–6.5) per 100 person‐years between 2014 and 2016. Model‐estimated HIV incidence in couple sub‐types where the male partner remained undiagnosed, diagnosed but virally unsuppressed, or virally suppressed throughout the simulation were also broadly consistent with empirical estimates in these subgroups [18, 22, 26]. Notably, because interventions were implemented only after an ANC visit that occurred halfway through 40 full weeks of gestation [15, 16], substantial transmission in the model occurred prior to intervention implementation, and transmission reductions became more apparent during the post‐partum period than during pregnancy. These findings suggest that efforts to reach pregnant people and their partners with HIV prevention services earlier in pregnancy, along with sustained adherence support that extends through lactation/breastfeeding, could optimize HIV incidence reductions.

Our model quantifies potential reductions in maternal HIV acquisition attainable at defined intervention uptake and adherence levels, without specifying how the interventions would be delivered or delineating the full range of considerations (such as any potential for intimate partner violence) that would require careful attention in their implementation. For example, when we estimate transmission reductions attainable if PrEP were provided to ANC patients whose partners have HIV infection (diagnosed or not), we do not specify how such patients would be identified. One approach to reaching all such people would be to provide PrEP to all HIV‐negative ANC patients with partners not known to be HIV–negative; however, such an approach would require PrEP provision to large numbers of patients, including some who might face a negligible risk of HIV acquisition. Approaches that rely on risk stratification to identify ANC patients most likely to benefit from PrEP could potentially minimize inefficiencies in practice. Similarly, we do not specify the mechanisms by which long‐term intervention adherence might be achieved, but (for example) our main analysis assumption of sustained PrEP adherence may approximate conditions achievable with long‐acting injectable PrEP [31].

We chose to focus on Malawi and Zambia due to their high HIV burdens and our ongoing HIV prevention work among ANC patients and their partners in these countries [10, 11, 12, 13, 14]. While specific quantitative results are most applicable to the populations of these countries, we expect that our findings are broadly generalizable to other eastern and southern African settings with similar patterns of sexual behaviour, HIV status awareness, viral suppression and ANC utilization. We also note that we relied on numerous simplifying assumptions for tractability. In particular, we did not consider transmissions occurring outside of the modelled steady partnerships, nor did we consider the potential effects of covariates such as age, male circumcision, concomitant sexually transmitted infections or CD4^+^ T‐cell count on coital frequencies or HIV transmission probabilities. Additionally, we assumed that PrEP initiation occurred only in the context of ANC, that incident diagnosis and treatment initiation among male partners only occurred through ANC‐prompted uptake of HIV services, and that PrEP and ART uptake was non‐differential with respect to sexual behaviour or other covariates. Furthermore, although our main findings were robust in a range of sensitivity analyses probing assumptions about intervention effectiveness, there were some scenarios—such as earlier or later ANC initiation and/or male partner engagement—that we did not explore. In general, we expect that higher real‐world effectiveness or earlier uptake of any given intervention would produce greater prevention impacts than those estimated, whereas lower effectiveness or later uptake would result in lesser impacts. We also expect that in settings where the introduction of HIV from outside partnerships is common for pregnant and lactating/breastfeeding people, female PrEP in particular would have greater overall impacts on maternal HIV incidence than those estimated. Finally, while the growing contribution of maternal HIV acquisition during pregnancy or lactation/breastfeeding to vertical HIV transmission [3] suggests that effective interventions against maternal HIV incidence during this period will have substantial downstream effects on paediatric infections, quantifying reductions in vertical HIV transmission was outside the scope of this study. We consider this aspect to be an important direction for future work.

## 5 CONCLUSIONS

Our model suggests that combination HIV prevention approaches could substantially reduce heterosexual HIV acquisition during pregnancy and lactation/breastfeeding in eastern and southern Africa. If scaled to target levels associated with transmission reductions of ∼45% in our model, these interventions could avert ∼54,000 of the estimated 120,000 maternal HIV infections acquired annually in UNAIDS focus countries [3]. Our modelling results, along with recent studies [13, 28] indicating that target uptake levels are attainable, suggest that extending ANC‐associated interventions towards primary HIV prevention for pregnant and lactating/breastfeeding people can improve maternal health and confront one of the final remaining barriers to ending vertical HIV transmission. Of note, our finding that substantial maternal HIV acquisition occurs before the first ANC visit indicates that interventions acting earlier in pregnancy—possibly in the context of pre‐conception planning or HIV testing outside of ANC settings—may offer important opportunities for optimizing both horizontal and vertical HIV prevention during pregnancy and lactation/breastfeeding.

## AUTHORS’ CONTRIBUTIONS

BHC, WM and KAP conceptualized the study. KAP designed and constructed the mathematical model, conducted all analyses and wrote the first draft of the article. LAG, NER, WM and BHC helped KAP to identify model inputs and select initial model scenarios. All authors contributed to the interpretation of results and manuscript revisions, and all authors approved the final manuscript. BHC and WM secured funding for the study.

## COMPETING INTERESTS

KRM has received grant support from the Bill & Melinda Gates Foundation, Gilead Sciences and Ridgeback Biotherapeutics. LAG reports consulting fees from UNICEF.

## FUNDING

This study was funded by the National Institute of Allergy and Infectious Diseases (NIAID) through award R01 AI131060. Additional investigator, trainee and administrative support was provided by NIAID (P30 AI050410, K24 AI120796), the Fogarty International Center (D43 TW009340, D43 TW010060) and NIMH (P30 MH062294).

## Supporting information


**Figure S1**: **Estimated intervention impact across range of scenarios with modified base‐case of 60% male ART initiation/re‐initiation**. Shaded region (note different scale vs. Fig. 2 and Fig. S2) represents the percentage of within‐couple, male‐to‐female infections averted during the specified intervals of pregnancy and lactation/breastfeeding (compared to base‐case conditions) according to the proportion of HIV‐negative female patients starting PrEP at the first ANC visit (horizontal axis) and the proportion of virologically unsuppressed, HIV‐diagnosed male partners initiating/re‐initiating suppressive ART after the first ANC visit (vertical axis) in scenarios where (left to right): 45% of partners become newly diagnosed through testing (base case), 65% of partners become newly diagnosed through testing, and 85% of partners become newly diagnosed through testing. Because all interventions begin at or after ANC initiation, which occurs after the end of early (first‐trimester) pregnancy, the impact of all interventions in the first 91 days is zero (and thus not shown).
**Figure S2**: **Estimated intervention impact across range of scenarios with modified base‐case of 80% male ART initiation/re‐initiation**. Shaded region represents the percentage of within‐couple, male‐to‐female infections averted during the specified intervals of pregnancy and lactation/breastfeeding (compared to base‐case conditions) according to the proportion of HIV‐negative female patients starting PrEP at the first ANC visit (horizontal axis) and the proportion of virologically unsuppressed, HIV‐diagnosed male partners initiating/re‐initiating suppressive ART after the first ANC visit (vertical axis) in scenarios where (left to right): 45% of partners become newly diagnosed through testing (base case), 65% of partners become newly diagnosed through testing, and 85% of partners become newly diagnosed through testing. Because all interventions begin at or after ANC initiation, which occurs after the end of early (first‐trimester) pregnancy, the impact of all interventions in the first 91 days is zero (and thus not shown).
**Figure S3**: **Estimated intervention impact across range of scenarios with constant per‐act transmission probability of 0.002**. Shaded region (note different scale vs. Fig. 2 and Figs. S1, S2) represents the percentage of within‐couple, male‐to‐female infections averted during the specified intervals of pregnancy and lactation/breastfeeding (compared to base‐case conditions) according to the proportion of HIV‐negative female patients starting PrEP at the first ANC visit (horizontal axis) and the proportion of virologically unsuppressed, HIV‐diagnosed male partners initiating/re‐initiating suppressive ART after the first ANC visit (vertical axis) in scenarios where (left to right): 45% of partners become newly diagnosed through testing (base case), 65% of partners become newly diagnosed through testing, and 85% of partners become newly diagnosed through testing. Because all interventions begin at or after ANC initiation, which occurs after the end of early (first‐trimester) pregnancy, the impact of all interventions in the first 91 days is zero (and thus not shown).

## Data Availability

Published estimates used to populate and parameterize the model are provided within the article. No primary data were collected for this analysis.

## References

[1] Astawesegn FH , Stulz V , Conroy E , Mannan H . Trends and effects of antiretroviral therapy coverage during pregnancy on mother‐to‐child transmission of HIV in sub‐Saharan Africa. Evidence from panel data analysis. BMC Infect Dis. 2022;22:134. 35135474 10.1186/s12879-022-07119-6 PMC8822759

[2] Graybill LA , Kasaro M , Freeborn K , Walker JS , Poole C , Powers KA , et al. Incident HIV among pregnant and breastfeeding women in sub‐Saharan Africa: a systematic review and meta‐analysis. AIDS. 2020;34(5):761–76. 32167990 10.1097/QAD.0000000000002487 PMC7275092

[3] Joint United Nations Programme for HIV/AIDS . Start Free, Stay Free, AIDS Free: final report on 2020 targets. Accessed January 20, 2022. https://www.unaids.org/sites/default/files/media_asset/2021_start‐free‐stay‐free‐aids‐free‐final‐report‐on‐2020‐targets_en.pdf

[4] Moran NF , Moodley J . The effect of HIV infection on maternal health and mortality. Int J Gynecol Obstet. 2012;119:S26–9. 10.1016/j.ijgo.2012.03.011 22889550

[5] Hoffman RF , Newhouse C , Chu B , Stringer JSA , Currier JS . Non‐communicable diseases in pregnant and postpartum women living with HIV: implications for health throughout the life course. Curr HIV/AIDS Rep. 2021;18:73–86. 33400169 10.1007/s11904-020-00539-6

[6] Mellins CA , Chu C , Malee K , Allison S , Smith R , Harris L , et al. Adherence to antiretroviral treatment among pregnant and postpartum HIV‐infected women. AIDS Care. 2008;20(8):958–68. 18608073 10.1080/09540120701767208

[7] Suandi D , Williams P , Bhattacharya S . Does involving male partners in antenatal care improve healthcare utilisation? Systematic review and meta‐analysis of the published literature from low‐ and middle‐income countries. Int Health. 2020;12(5):484–98. 31613327 10.1093/inthealth/ihz073 PMC11701106

[8] Pintye J , Beima‐Sofie KM , Kimemia G , Kimemia G , Ngure K , Trinidad SB , et al. “I did not want to give birth to a child who has HIV”: experiences using PrEP during pregnancy among HIV‐uninfected Kenyan women in HIV‐serodiscordant couples. J Acquir Immune Defic Syndr. 2017;76(3):259–65. 28777265 10.1097/QAI.0000000000001516 PMC5634917

[9] Chi BH , Rosenberg NE , Mweemba O , Powers KA , Zimba C , Maman S , et al. Involving both parents in HIV prevention during pregnancy and breastfeeding. Bull World Health Organ. 2018;96(1):69–71. 29403103 10.2471/BLT.17.200139 PMC5791874

[10] Hershow RB , Zimba CC , Mweemba O , Chibwe KF , Phanga T , Dunda W , et al. Perspectives on HIV partner notification, partner self‐testing and partner home‐based HIV testing by pregnant and postpartum women in antenatal settings: a qualitative analysis in Malawi and Zambia. J Int AIDS Soc. 2019;22(Suppl 3):e25293. 31321884 10.1002/jia2.25293 PMC6639664

[11] Zimba C , Maman S , Rosenberg NE , Mutale W , Mweemba O , Dunda W , et al. The landscape for HIV pre‐exposure prophylaxis during pregnancy and breastfeeding in Malawi and Zambia: a qualitative study. PLoS One. 2019;14(10):e0223487. 31584987 10.1371/journal.pone.0223487 PMC6777778

[12] Mweemba O , Zimba C , Chi BH , Chibwe KF , Dunda W , Freeborn K , et al. Contextualising men's role and participation in PMTCT programmes in Malawi and Zambia: a hegemonic masculinity perspective. Glob Public Health. 2022;17(9):2081–94. 34375155 10.1080/17441692.2021.1964559

[13] Mutale W , Freeborn K , Graybill LA , Lusaka MM , Mollan KR , Mweemba O , et al. Addition of HIV self‐test kits to partner notification services to increase HIV testing of male partners of pregnant women in Zambia: two parallel randomized trials. Lancet Glob Health. 2021;9(1):e1719–29. 34735862 10.1016/S2214-109X(21)00393-4 PMC8644317

[14] Saidi F , Mutale W , Freeborn K , Rosenberg NE , Graybill LA , Maman S , et al. Combination adherence strategy to support HIV antiretroviral therapy and pre‐exposure prophylaxis adherence during pregnancy and breastfeeding: protocol for a pair of pilot randomized trials. BMJ Open. 2021;11(6):e046032. 10.1136/bmjopen-2020-046032 PMC8246367 34193491

[15] National Statistical Office [Malawi] and ICF . Malawi Demographic and Health Survey 2015–2016. Zomba, Malawi, and Rockville, MD: NSO and ICF; 2017.

[16] Zambia Statistics Agency, Ministry of Health Zambia, and ICF . Zambia demographic and health survey. Lusaka, Zambia, and Rockville, MD: Zambia Statistics Agency, Ministry of Health, and ICF; 2018.

[17] Joint United Nations Programme on HIV/AIDS (UNAIDS) . UNAIDS Data 2020. Geneva: 2020. 12349391

[18] Thomson KA , Hughes J , Baeten JM , John‐Stewart G , Celum C , Cohen CR , et al. Increased risk of HIV acquisition among women throughout pregnancy and during the postpartum period: a prospective per‐coital‐act analysis among women with HIV‐infected partners. J Infect Dis. 2018;218:16–25. 29514254 10.1093/infdis/jiy113 PMC5989601

[19] Awopegba OE , Kalu A , Ahinkorah BO , Siedu A‐A , Ajayi AI . Prenatal care coverage and correlates of HIV testing in sub‐Saharan Africa: insight from demographic and health surveys of 16 countries. PLoS One. 2020;15(11):e0242001. 33166351 10.1371/journal.pone.0242001 PMC7652338

[20] Eshleman SH , Wilson EA , Zhang XC , Ou S‐S , Piwowar‐Manning E , Eron JJ , et al. Virologic outcomes in early antiretroviral treatment: HPTN 052. HIV Clin Trials. 2017;18(3):100–9. 28385131 10.1080/15284336.2017.1311056 PMC5633001

[21] Cahn P , Sierra Madero J , Arribas JR , Antinori A , Ortiz R , Clarke AE , et al. Three‐year durable efficacy of dolutegravir plus lamivudine in antiretroviral therapy‐naïve adults with HIV infection. AIDS. 2022;36(1):39–48. 34534138 10.1097/QAD.0000000000003070 PMC8654248

[22] Wawer MJ , Gray RH , Sewankambo NK , Serwadda D , Li X , Laeyendecker O , et al. Rates of HIV‐1 transmission per coital act, by stage of HIV‐1 infection, in Rakai, Uganda. J Infect Dis. 2005;191(9):1403–9. 15809897 10.1086/429411

[23] Powers KA , Poole C , Pettifor AE , Cohen MS . Rethinking the heterosexual infectivity of HIV‐1: a systematic review and meta‐analysis. Lancet Infect Dis. 2008;8(9):553–63. 18684670 10.1016/S1473-3099(08)70156-7 PMC2744983

[24] Marks G , Crepaz N , Senterfitt JW , Janssen RS . Meta‐analysis of high‐risk sexual behavior in persons aware and unaware they are infected with HIV in the United States. J Acquir Immune Defic Syndr. 2005;39(4):446–53. 16010168 10.1097/01.qai.0000151079.33935.79

[25] Baeten JM , Donnell D , Ndase P , Mugo NR , Campbell JD , Wangisi J , et al. Antiretroviral prophylaxis for HIV prevention in heterosexual men and women. New Engl J Med. 2012;367(5):399–410. 22784037 10.1056/NEJMoa1108524 PMC3770474

[26] Cohen MS , Chen YQ , McCauley M , Gamble T , Hosseinipour MC , Kumarasamy N , et al. Prevention of HIV‐1 infection with early antiretroviral therapy. N Engl J Med. 2011;365(6):493–505. 21767103 10.1056/NEJMoa1105243 PMC3200068

[27] Joint United Nations Programme on HIV/AIDS (UNAIDS) . 2025 AIDS Targets. Accessed November 19, 2021. https://aidstargets2025.unaids.org/

[28] Kinuthia J , Pintye J , Abuna F , Mugwanya KF , Lagat H , Onyango D , et al. Pre‐exposure prophylaxis uptake and early continuation among pregnant and post‐partum women within maternal and child health clinics in Kenya: results from an implementation programme. Lancet HIV. 2020;7:38–48. 10.1016/S2352-3018(19)30335-2 PMC11498332 31813837

[29] Blaser N , Salazar Vizcaya L , Estill J , Zahnd C , Kalesan B , Egger M , et al. Gems: an R package for simulating from disease progression models. J Stat Soft. 2015;64(10):1–22. PMC4458858 26064082

[30] Joseph Davey D , Bekker L‐G , Gomba Y , Coates T , Myer L , Johnson LF . Modelling the potential impact of providing pre‐exposure prophylaxis (PrEP) in pregnant and breastfeeding women in South Africa. AIDS. 2019;33(8):1391–5. 30950882 10.1097/QAD.0000000000002221 PMC6561341

[31] Moyo E , Murewanhema MG , Dzinamarira T . Long‐acting injectable drugs for HIV‐1 pre‐exposure prophylaxis: considerations for Africa. Trop Med Infect Dis. 2022;7(8):154. 36006246 10.3390/tropicalmed7080154 PMC9414191

