# Supplementary material for: Combination HIV prevention during pregnancy and the post‐partum period in Malawi and Zambia: a mathematical modelling analysis
Source: J Int AIDS Soc. 2023 Jul 4;26(7):e26128. doi: 10.1002/jia2.26128 (PMC10320044; doi:10.1002/jia2.26128)
Supplement: Supplementary file 1 — Figure S1: Estimated intervention impact across range of scenarios with modified base‐case of 60% male ART initiation/re‐initiation. Shaded region (note different scale vs. Fig. 2 and Fig. S2) represents the percentage of within‐couple, male‐to‐female infections averted during the specified intervals of pregnancy and lactation/breastfeeding (compared to base‐case conditions) according to the proportion of HIV‐negative female patients starting PrEP at the first ANC visit (horizontal axis) and the proportion of virologically unsuppressed, HIV‐diagnosed male partners initiating/re‐initiating suppressive ART after the first ANC visit (vertical axis) in scenarios where (left to right): 45% of partners become newly diagnosed through testing (base case), 65% of partners become newly diagnosed through testing, and 85% of partners become newly diagnosed through testing. Because all interventions begin at or after ANC initiation, which occurs after the end of early (first‐trimester) pregnancy, the impact of all interventions in the first 91 days is zero (and thus not shown). Figure S2: Estimated intervention impact across range of scenarios with modified base‐case of 80% male ART initiation/re‐initiation. Shaded region represents the percentage of within‐couple, male‐to‐female infections averted during the specified intervals of pregnancy and lactation/breastfeeding (compared to base‐case conditions) according to the proportion of HIV‐negative female patients starting PrEP at the first ANC visit (horizontal axis) and the proportion of virologically unsuppressed, HIV‐diagnosed male partners initiating/re‐initiating suppressive ART after the first ANC visit (vertical axis) in scenarios where (left to right): 45% of partners become newly diagnosed through testing (base case), 65% of partners become newly diagnosed through testing, and 85% of partners become newly diagnosed through testing. Because all interventions begin at or after ANC initiation, which occurs after the end of [file JIA2-26-e26128-s001.docx]

**SUPPLEMENTAL MATERIAL**

**FOR**

**Combination HIV Prevention during Pregnancy and the Post-Partum Period in Malawi and Zambia:**

**A Mathematical Modeling Analysis**

Kimberly A. Powers, Wilbroad Mutale, Nora E. Rosenberg, Lauren A. Graybill, Katie R. Mollan,

Kellie Freeborn, Friday Saidi, Suzanne Maman, Priscilla L. Mulenga, Andreas Jahn, Rose K. Nyirenda,

Jeffrey S. A. Stringer, Sten H. Vermund, Benjamin H. Chi

***Supplemental Methods: Model Description***

***Defining Sub-Cohorts***

As described in the main text, we developed a multi-state model to describe male-to-female HIV transmission during pregnancy and lactation/breastfeeding within steady, heterosexual pairs of men with HIV and women without HIV at pregnancy start. In this model, we delineated 12 couple types (or “sub-cohorts”) according to: a) the male partner’s baseline diagnosis status (HIV-diagnosed vs. not HIV-diagnosed), b) the male partner’s baseline viral suppression status if already HIV-diagnosed (virologically suppressed vs. not virologically suppressed), c) the male partner’s eventual HIV diagnosis status after female antenatal care (ANC) presentation if not HIV-diagnosed at baseline (will become HIV-diagnosed after ANC vs. will not become HIV-diagnosed after ANC), d) the male partner’s eventual viral suppression status after female ANC presentation if already diagnosed at baseline or newly diagnosed after female ANC presentation (will initiate ART and become virally suppressed vs. will not initiate ART and become virally suppressed), and e) the female partner’s eventual PrEP status upon ANC entry (will initiate PrEP vs. will not initiate PrEP):

|  | **At Baseline (Pregnancy Start):** | | **During Follow-Up:** | | |
| --- | --- | --- | --- | --- | --- |
| **Couple Type** | **Male HIV**  **Diagnosis** | **Male Viral Suppression** | **Male Incident HIV Diagnosis** | **Male Incident Suppression** | **Female**  **Incident PrEP** |
| 1 | Not diagnosed | Not suppressed | No | No | No |
| 2 | Not diagnosed | Not suppressed | Yes | No | No |
| 3 | Not diagnosed | Not suppressed | Yes | Yes | No |
| 4 | Not diagnosed | Not suppressed | No | No | Yes |
| 5 | Not diagnosed | Not suppressed | Yes | No | Yes |
| 6 | Not diagnosed | Not suppressed | Yes | Yes | Yes |
| 7 | Diagnosed | Not suppressed | N/A | No | No |
| 8 | Diagnosed | Not suppressed | N/A | Yes | No |
| 9 | Diagnosed | Not suppressed | N/A | No | Yes |
| 10 | Diagnosed | Not suppressed | N/A | Yes | Yes |
| 11 | Diagnosed | Suppressed | N/A | N/A | No |
| 12 | Diagnosed | Suppressed | N/A | N/A | Yes |

This set of couple types was chosen to include major pair sub-types with respect to diagnosis/ART/PrEP status while maintaining model tractability/interpretability. As such, this set is not exhaustive and excludes some (likely smaller) subsets of real-world couples (in whom, for example, male partners initiate ART but do not become virologically suppressed).

***Calculating Cumulative Incidence by Sub-Cohort and Timeframe***

We then used the gems package^1^ in R to calculate cumulative HIV incidence and corresponding 95% confidence intervals in each sub-cohort (each with a sample size of 1 million couples) through each of the four timeframes specified in the main text (early pregnancy, late pregnancy, early post-partum, late post-partum).

We specify nine states in the model according to the female partner’s HIV status and the time sub-interval within pregnancy and breastfeeding. The first eight states are defined according to the time elapsed, with instantaneous transitions from one state to the next at the times specified (in days) below for couples in whom transmission had not yet occurred:


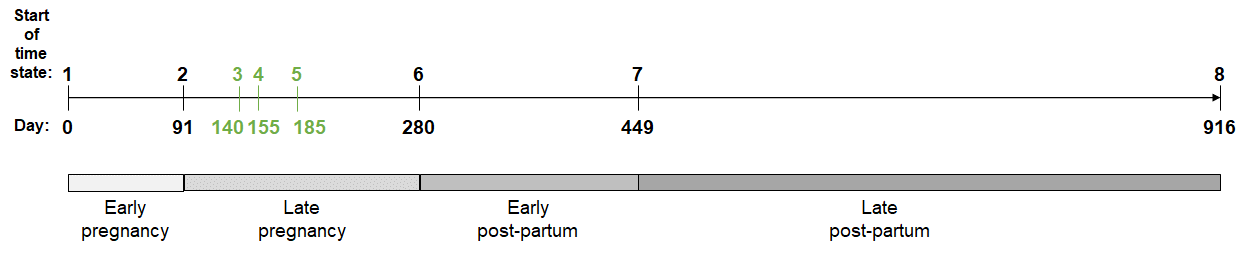


In the above figure, start times for each state labeled in black text correspond to pregnancy/post-partum periods.^2^ The start time for state 3 corresponds to the time of the first ANC visit (when it was assumed that female PrEP use could begin), the start time for state 4 corresponds to 15 days after the first ANC visit (when it was assumed that male HIV testing and/or ART initiation could occur), and the start time for state 5 corresponds to 30 days after the possible time of post-ANC male ART initiation (when it was assumed that viral suppression was achieved).

The ninth state represents the absorbing HIV-concordant-positive state that is reached when within-couple HIV transmission occurs. The corresponding hazard matrix for each sub-cohort is as follows:

|  | **Hazard for transition to concordant-positive state via intra-couple HIV transmission:** | | | | | | |
| --- | --- | --- | --- | --- | --- | --- | --- |
| **Sub-cohort** | **1 → 9** | **2 → 9** | **3 → 9** | **4 → 9** | **5 → 9** | **6 → 9** | **7 → 9** |
| 1 | c_1_β_1_ | c_2_β_2_ | c_2_β_2_ | c_2_β_2_ | c_2_β_2_ | c_3_β_3_ | c_4_β_4_ |
| 2 | c_1_β_1_ | c_2_β_2_ | c_2_β_2_ | d(c_2_β_2_) | d(c_2_β_2_) | d(c_3_β_3_) | d(c_4_β_4_) |
| 3 | c_1_β_1_ | c_2_β_2_ | c_2_β_2_ | d(c_2_β_2_) | dv(c_2_β_2_) | dv(c_3_β_3_) | dv(c_4_β_4_) |
| 4 | c_1_β_1_ | c_2_β_2_ | p(c_2_β_2_) | p(c_2_β_2_) | p(c_2_β_2_) | p(c_3_β_3_) | p(c_4_β_4_) |
| 5 | c_1_β_1_ | c_2_β_2_ | p(c_2_β_2_) | pd(c_2_β_2_) | pd(c_2_β_2_) | pd(c_3_β_3_) | pd(c_4_β_4_) |
| 6 | c_1_β_1_ | c_2_β_2_ | p(c_2_β_2_) | pd(c_2_β_2_) | pdv(c_2_β_2_) | pdv(c_3_β_3_) | pdv(c_4_β_4_) |
| 7 | d(c_1_β_1_) | d(c_2_β_2_) | d(c_2_β_2_) | d(c_2_β_2_) | d(c_2_β_2_) | d(c_3_β_3_) | d(c_4_β_4_) |
| 8 | d(c_1_β_1_) | d(c_2_β_2_) | d(c_2_β_2_) | d(c_2_β_2_) | dv(c_2_β_2_) | dv(c_3_β_3_) | dv(c_4_β_4_) |
| 9 | d(c_1_β_1_) | d(c_2_β_2_) | dp(c_2_β_2_) | dp(c_2_β_2_) | dp(c_2_β_2_) | dp(c_3_β_3_) | dp(c_4_β_4_) |
| 10 | d(c_1_β_1_) | d(c_2_β_2_) | dp(c_2_β_2_) | dp(c_2_β_2_) | dpv(c_2_β_2_) | dpv(c_3_β_3_) | dpv(c_4_β_4_) |
| 11 | dv(c_1_β_1_) | dv(c_2_β_2_) | dv(c_2_β_2_) | dv(c_2_β_2_) | dv(c_2_β_2_) | dv(c_3_β_3_) | dv(c_4_β_4_) |
| 12 | dv(c_1_β_1_) | dv(c_2_β_2_) | dvp(c_2_β_2_) | dvp(c_2_β_2_) | dvp(c_2_β_2_) | dvp(c_3_β_3_) | dvp(c_4_β_4_) |

where c_1_, c_2_, c_3_, and c_4_ represent condomless coital frequency (acts/day) in the early pregnancy, late pregnancy, early post-partum, and late-postpartum periods, respectively, if the male partner is undiagnosed; β_1_, β_2_, β_3_, and β_4_ represent transmission probabilities per condomless coital act in those same periods if the male partner is not virally suppressed and the female partner is not on PrEP; d = the relative coital frequency if the male partner is diagnosed (vs. undiagnosed); v = the relative per-act transmission probability if the male partner is diagnosed and virally suppressed (vs. diagnosed and not virally suppressed); and p = the relative per-act transmission probability if the female partner is on PrEP.

Values used in the model for β_1_ - β_4_ (see Table 1 in main text) were drawn directly from an analysis of HIV acquisition risk during pregnancy and breastfeeding among HIV-negative women with male partners known to be living with HIV in seven African countries.^2^ Values for c_1_ – c_4_ were based on overall coital frequencies (i.e., including both condomless and condom-protected acts) reported across the specified sub-intervals in this same analysis,^2^ under the assumption that the overall coital frequency in these couples with a known HIV-positive partner would approximate *condomless* coital frequency in couples where the male partner with HIV is unaware of his infection. This assumption aligns with low levels of within-partnership condom use reported in Rakai, Uganda by Wawer *et al*.^3^

***Calculating Total Transmission Events and Transmissions Averted***

After calculating the cumulative HIV incidence and corresponding 95% confidence intervals within each sub-cohort of 1 million couples, we then calculated the expected number of HIV transmissions at each of the four endpoints in populations of 12 million couples with different distributions of couples across sub-cohorts (as defined by the specific model scenario). We show as examples the calculations (under main-analysis conditions) through 280 days for the “base-case” and “target” scenarios (for all three interventions) defined in the main text:

| **Couple Type** | **Allocation Formula*** | **Number of Couples:**  **Base-Case Scenario^†^** | **Number of Couples:**  **Target**  **Scenario^‡^** | **Cumulative Incidence at 280 days^¶^** | **Estimated Transmissions by 280 days:**  **Base-Case**** | **Estimated Transmissions by 280 days:**  **Target**** |
| --- | --- | --- | --- | --- | --- | --- |
| 1 | (1-δ)(1-θ)(1-ρ)N | 858,000 | 46,800 | 0.088 | 75,888.384 | 4,139.366 |
| 2 | (1-δ)θ(1-τ)(1-ρ)N | 175,500 | 88,920 | 0.069 | 12,037.194 | 6,098.845 |
| 3 | (1-δ)θτ(1-ρ)N | 526,500 | 800,280 | 0.055 | 29,206.53 | 44,393.93 |
| 4 | (1-δ)(1-θ)ρN | 0 | 31,200 | 0.057 | 0 | 1,777.8696 |
| 5 | (1-δ)θ(1-τ)ρN | 0 | 59,280 | 0.052 | 0 | 3079.892 |
| 6 | (1-δ)θτρN | 0 | 533,520 | 0.049 | 0 | 25,908.80 |
| 7 | δ(1-ω)(1-τ)(1-ρ)N | 600,300 | 144,072 | 0.043 | 25,519.954 | 6124.789 |
| 8 | δ(1-ω)τ(1-ρ)N | 1,800,900 | 1,296,648 | 0.029 | 52,314.34 | 37,666.33 |
| 9 | δ(1-ω)(1-τ)ρN | 0 | 96,048 | 0.027 | 0 | 2,614.715 |
| 10 | δ(1-ω)τρN | 0 | 864,432 | 0.024 | 0 | 20,520.75 |
| 11 | δω(1-ρ)N | 8,038,800 | 4,823,280 | 0.002 | 14,108.09 | 8,464.856 |
| 12 | δωρN | 0 | 3,215,520 | 0.001 | 0.0 | 3,566.012 |
| Total | N | 12,000,000 | 12,000,000 |  | 209,074.5 | 164,356.2 |

* Where δ = proportion of male partners (all of whom have HIV infection) who are already HIV-diagnosed at pregnancy start, ω = proportion of HIV-diagnosed male partners already suppressed at pregnancy start; θ = proportion of undiagnosed male partners who will be diagnosed after female ANC presentation; τ = proportion of unsuppressed males (both newly and previously diagnosed) who will start suppressive ART after ANC presentation, and ρ = proportion of females who will start PrEP at ANC.

^†^ Calculated with allocation formula and base-case values of δ = 0.87, ω = 0.77, θ = 0.45, τ = 0.75, ρ = 0.0.

^‡^ Calculated with allocation formula and “target scenario” values of δ = 0.87, ω = 0.77, θ = 0.95, τ = 0.90, ρ = 0.40.

¶ Proportion of couples in sub-cohort in whom transmission occurs through specified endpoint, as calculated with multi-state model (see also Table 2 in main text).

** Calculated by multiplying cohort-specific cumulative incidence by cohort- and scenario-specific number of couples. Similarly, we calculated the lower and upper confidence limits for the number of transmissions in a given sub-cohort by multiplying the cumulative incidence confidence limits by the scenario-specific number of couples in the sub-cohort.

We calculated the percentage of infections averted for a given scenario (vs. base-case) at a given time point with the following equation:

$$\% transmissions averted = 100*\left[ 1-\frac{total transmission events\left( intervention scenario \right)}{total transmission events \left( base case \right)} \right]$$

To calculate the lower and upper 95% confidence limits corresponding to the percentage of transmissions averted, we first calculated the squared standard error for the number of transmissions in each sub-cohort as the upper minus the lower 95% confidence limit on estimated transmissions within the cohort for a given time period and scenario, divided by 3.92 and then squared. We then summed the squared standard errors across sub-cohorts and took the square root, arriving at the standard error on the total number of transmissions (i.e., across all sub-cohorts) in a given scenario at a given time point. Next, we calculated the standard error for the percentage of transmissions averted^4^ as:

$$SE\left( \% transmissions averted \right)=\frac{{events}_{s}}{{events}_{0}}*\sqrt{\frac{{SE}_{s}^{2}}{{events}_{s}^{2}}+\frac{{SE}_{0}^{2}}{{events}_{0}^{2}}}$$

where *SE_s_* = the standard error on total transmission events (across sub-cohorts) in scenario *s* for a given time period, with *s*=0 representing the base-case scenario. Finally, we calculated the lower and upper confidence limits on the percentage of transmissions averted by subtracting and adding the product of 1.96 and SE(% transmissions averted), respectively, to the estimated % of transmissions averted.

***Supplemental Methods: References***

1. Blaser N, Salazar Vizcaya L, Estill J, Zahnd C, Kalesan B, Egger M, et al. gems: An R package for simulating from disease progression models. *J Stat Soft* 2015; 64(10): 1-22
2. Thomson KA, Hughes J, Baeten JM, John-Stewart G, Celum C, Cohen CR, et al. Increased risk of HIV acquisition among women throughout pregnancy and during the postpartum period: a prospective per-coital-act analysis among women with HIV-infected partners. *J Infect Dis* 2018; 218:16-25
3. Wawer MJ, Gray RH, Sewankambo NK, Serwadda D, Li X, Laeyendecker O, et al. Rates of HIV-1 transmission per coital act, by stage of HIV-1 infection, in Rakai, Uganda. *J Infect Dis* 2005; 191(9):1403-9.
4. United States Census Bureau. Percent Changes. <https://www2.census.gov/programs-surveys/acs/tech_docs/accuracy/percchg.pdf>

***Supplemental Results: Sensitivity Analysis with Base-Case Male ART Uptake = 60%***


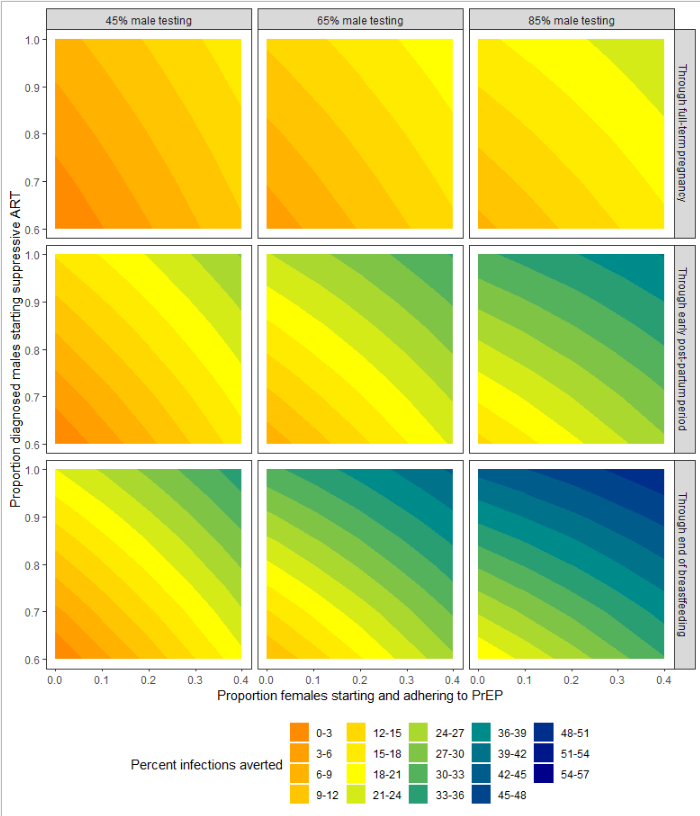


**Supplemental Figure S1. Estimated intervention impact across range of scenarios with modified base-case of 60% male ART initiation/re-initiation.** Shaded region (note different scale vs. Fig. 2 and Fig. S2) represents the percentage of within-couple, male-to-female infections averted during the specified intervals of pregnancy and lactation/breastfeeding (compared to base-case conditions) according to the proportion of HIV-negative female patients starting PrEP at the first ANC visit (horizontal axis) and the proportion of virologically unsuppressed, HIV-diagnosed male partners initiating/re-initiating suppressive ART after the first ANC visit (vertical axis) in scenarios where (left to right): 45% of partners become newly diagnosed through testing (base case), 65% of partners become newly diagnosed through testing, and 85% of partners become newly diagnosed through testing. Because all interventions begin at or after ANC initiation, which occurs after the end of early (first-trimester) pregnancy, the impact of all interventions in the first 91 days is zero (and thus not shown).

***Supplemental Results: Sensitivity Analysis with Base-Case Male ART Uptake = 80%***


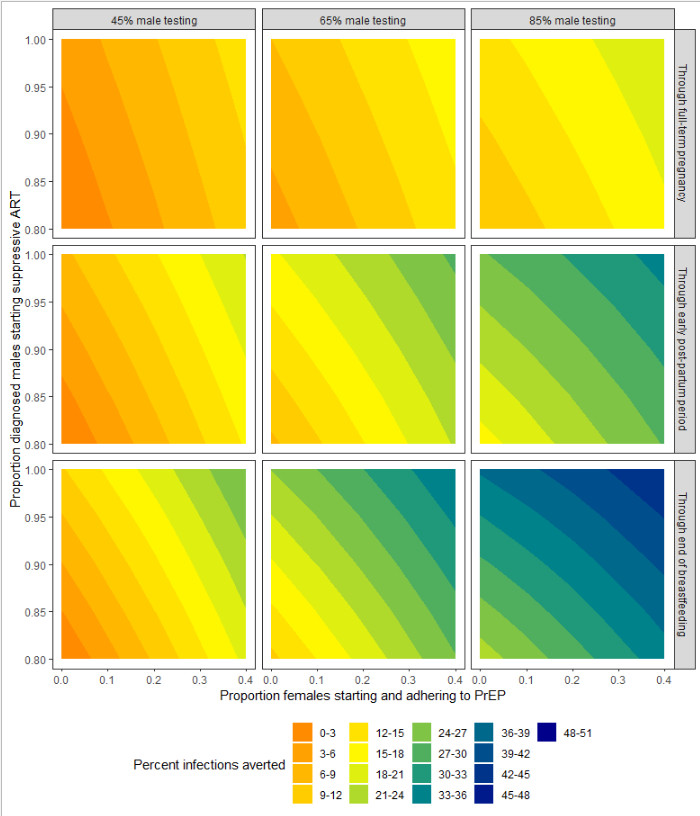


**Supplemental Figure S2. Estimated intervention impact across range of scenarios with modified base-case of 80% male ART initiation/re-initiation.** Shaded region represents the percentage of within-couple, male-to-female infections averted during the specified intervals of pregnancy and lactation/breastfeeding (compared to base-case conditions) according to the proportion of HIV-negative female patients starting PrEP at the first ANC visit (horizontal axis) and the proportion of virologically unsuppressed, HIV-diagnosed male partners initiating/re-initiating suppressive ART after the first ANC visit (vertical axis) in scenarios where (left to right): 45% of partners become newly diagnosed through testing (base case), 65% of partners become newly diagnosed through testing, and 85% of partners become newly diagnosed through testing. Because all interventions begin at or after ANC initiation, which occurs after the end of early (first-trimester) pregnancy, the impact of all interventions in the first 91 days is zero (and thus not shown).

***Supplemental Results: Sensitivity Analysis with Constant HIV Transmission Probability***


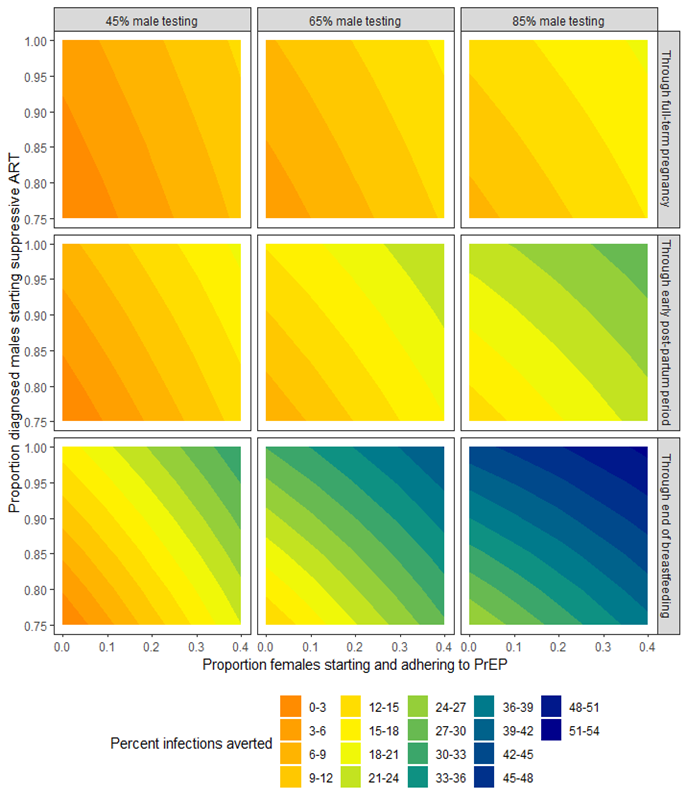


**Supplemental Figure S3. Estimated intervention impact across range of scenarios with constant per-act transmission probability of 0.002.** Shaded region (note different scale vs. Fig. 2 and Figs. S1, S2) represents the percentage of within-couple, male-to-female infections averted during the specified intervals of pregnancy and lactation/breastfeeding (compared to base-case conditions) according to the proportion of HIV-negative female patients starting PrEP at the first ANC visit (horizontal axis) and the proportion of virologically unsuppressed, HIV-diagnosed male partners initiating/re-initiating suppressive ART after the first ANC visit (vertical axis) in scenarios where (left to right): 45% of partners become newly diagnosed through testing (base case), 65% of partners become newly diagnosed through testing, and 85% of partners become newly diagnosed through testing. Because all interventions begin at or after ANC initiation, which occurs after the end of early (first-trimester) pregnancy, the impact of all interventions in the first 91 days is zero (and thus not shown).
